# The rodent models of arteriovenous fistula

**DOI:** 10.3389/fcvm.2024.1293568

**Published:** 2024-01-18

**Authors:** Yuxuan Li, Ke Hu, Yiqing Li, Chanjun Lu, Yi Guo, Weici Wang

**Affiliations:** ^1^ Department of Vascular Surgery, Union Hospital, Tongji Medical College, Huazhong University of Science and Technology, Wuhan, China; ^2^ Department of General Vascular Surgery, Wuhan No.1 Hospital & Wuhan Hospital of Traditional Chinese and Western Medicine, Wuhan, China; ^3^ Clinic Center of Human Gene Research, Union Hospital, Tongji Medical College, Huazhong University of Science and Technology, Wuhan, China; ^4^ Cardiovascular Center, Liyuan Hospital, Tongji Medical College, Huazhong University of Science and Technology, Wuhan, China

**Keywords:** arteriovenous fistula, chronic kidney disease, mice, rats, rodent model

## Abstract

Arteriovenous fistulas (AVFs) have long been used as dialysis access in patients with end-stage renal disease; however, their maturation and long-term patency still fall short of clinical needs. Rodent models are irreplaceable to facilitate the study of mechanisms and provide reliable insights into clinical problems. The ideal rodent AVF model recapitulates the major features and pathology of human disease as closely as possible, and pre-induction of the uremic milieu is an important addition to AVF failure studies. Herein, we review different surgical methods used so far to create AVF in rodents, including surgical suturing, needle puncture, and the cuff technique. We also summarize commonly used evaluations after AVF placement. The aim was to provide recent advances and ideas for better selection and induction of rodent AVF models. At the same time, further improvements in the models and a deeper understanding of AVF failure mechanisms are expected.

## 1 Introduction

With an aging population, the incidence and prevalence of chronic kidney disease (CKD) are increasing annually, and the number of patients requiring hemodialysis (HD) is growing exponentially (1, 2) According to clinical practice guidelines, arteriovenous fistulas (AVFs) are a crucial bridge used for HD access in patients with end-stage renal disease (ESRD) (3). Importantly, experts prefer using autologous AVFs for vascular access owing to their low possibility of developing infections and other non-thrombotic complications than AVF graft (4). However, the long-term patency of autologous AVFs is poor, and their application yet fails to meet clinical needs owing to early maturation failure, late stenosis, and formation of thrombosis (5, 6). Patients with complications and treatment related to vascular access account for nearly one-third of HD admissions (2). One main reason for AVF maturation failure is insufficient outward remodeling, which fails to adapt to changes in hemodynamics following arterialization (7). Another reason is excessive neointimal hyperplasia (NIH) within the fistula and thrombosis at the anastomosis, leading to luminal stenosis (8). Previous studies have shown that aggressive NIH was found in both pig models of AVF graft stenosis and stenotic venous segments in patients with early AVF failure (9, 10).

In general, researchers have focused on the pathogenesis of AVF failure and have conducted many studies. In depth exploration and experimentation with new drugs or vascular coatings is needed to obtain effective methods to improve the long-term patency and extend the duration of AVFs. An establishment of a good animal model to reproduce this particular pathophysiology is key to the rigour and success of subsequent studies. It was shown that multiple vascular biological pathways may be involved in causing the development of this pathologic change, including inflammation, oxidative stress, hypoxia, and altered hemodynamics (7). Large animals are anatomically and physically closer to human and can effectively demonstrate hemodynamic changes, which is a limitation of small animals. However, rodents are good choices in other respects and are irreplaceable due to their low cost, abundance of resources, large sample sizes, and maturity in genetic studies (11, 12). Another major advantage of the rodent AVF model is the rapid development of significant neointimal damage, thus facilitating short-term intervention studies (13, 14). Several rodent AVF models have been established using surgical manipulation; however, an ideal uniform standard is yet to be established for developing model methods.

In this review, we searched related articles in PubMed up to November 2022 using the following terms in titles and abstracts: (mouse OR rat OR rodent) AND (arteriovenous fistula). In this study, we described primarily used contents of AVF models and the need to pre-induce a CKD milieu in the AVF model for better disease mimicry. We then reviewed the evolutionary history of rodent AVF models, summarizing methods available to create AVF in rodents based on different surgical approaches, including general suturing, needle puncture, and the cuff technique. Finally, common evaluations after AVF placement are introduced. We hope that this review can provide recent advances and new hints for the selection and induction of rodent AVF models and can help deepen our understanding of existing studies on the molecular mechanisms of AVF.

## 2 Main study contents and requirements of AVF models

AVF is a lifeline for ESRD patients, and longer dialysis durations can last over a decade; however, the success rate of AVF creation and its durability still need to be improved (15). An ideal AVF is required to achieve an anticipated duration and fewer complications when patients require HD for more than 1 year (3). At present, most studies use AVF animal models to investigate the molecular mechanisms of the pathophysiologic processes associated with AVF failure.

There is a different understanding of AVF failure, depending on the stage of occurrence and cause. First, autologous AVFs usually takes 4–6 weeks after the procedure to mature; therefore, are not immediately available to use (3, 16). This clinical maturation process is accompanied by hemodynamic changes, mild venous dilatation, and vascular remodeling (17, 18). Endothelial injury, altered blood flow and shear stress activate myofibroblasts, fibroblasts, and immune cells along with an increase in pro-inflammatory cytokines and growth factors to promote the proliferation and migration of smooth muscle cells (SMCs) to the intimal layer (10, 19). Once vascular remodeling is unbalanced, excessive intimal hyperplasia causes early fistula maturation failure (20, 21). An imbalance in the regulation of extracellular matrix degradation and deposition in the vessel wall causes wall thickening during AVF maturation (18, 22). In contrast, a good patency rate of the fistula is required to meet functional needs. Similarly, NIH is the main identified etiology of poor patency at the venous limb or near anastomosis regions and attendant venous stenosis, which is also prone to thrombosis (23). Previous studies have suggested that the specific pathogenesis of AVF failure involves hypoxia (24), oxidative stress (25, 26), inflammation (27–29), uremia (30), and hemodynamics (31, 32). It has been demonstrated that vascular remodeling in fistulas characterized by high flow and low pressure is associated with upregulation of matrix metalloproteinases (MMPs; MMP-2 and MMP-9), reduced tissue inhibitors of MMPs, and collagen degradation (18). Castier et al. found that changes such as wall shear stress (WSS) induce elevated levels of oxidative stress and increase reactive oxygen species production by NADPH oxidase, which in turn activates MMPs to promote vascular remodeling (31). Platelet activation during endothelial injury causes the release of platelet-derived growth factor, stimulating the upregulation of the expression of a range of pro-inflammatory cytokines including tumor necrosis factor-α, which can mediate the proliferation and migration of SMCs (8). However, the specific molecular mechanisms remain to be explored further to develop effective coping strategies.

Complications following fistula treatment include ischemic neuropathy, edema, infection, hematoma, and subclavian steal syndrome, which have also been studied using AVF models, although they account for a small proportion (33–35). Furthermore, because AVF induces hyperdynamic circulation, the probability of adverse cardiovascular events increases, and the use of surgical aortocaval fistulas to simulate chronic cardiac load in rats has been reported (36). Carotid-jugular fistulas can be used to simulate altered cerebral blood flow in arteriovenous malformations, pathophysiology of microcirculatory changes, induction of venous hypertension (37, 38), and exploration of the role of AVF in “cardiorenal syndrome” (39, 40). Nevertheless, AVF failure due to stenosis and thrombosis remains a significant concern in basic research, based on its clinical value. Studies have demonstrated that the period of AVF maturation is longer in diabetic patients; therefore, studies related to AVF failure based on the specific disease background of diabetes are available (41, 42).

To establish a standard experimental animal model of AVF, certain basic requirements must be met. First, careful consideration must be given to animal welfare, and adherence to the “3Rs principles” of “replacement, reduction, and refinement” is necessary before conducting any animal research (43). Second, the AVF animal model should meet the following experimental requirements as far as possible: it should be able to simulate the arterialized vein process, have a high degree of similarity to human hemodynamics, be relatively easy to establish, and have a good balance between cost-effectiveness and sample size. Furthermore, AVF patency rates are important indicators of interest that help determine the timing of sample acquisition and intervention (Table 1).

**Table 1:** Examples of the rodent AVF models for studying AVF failure.

Animal species	Model methods		Study time points	Patency rates	Main characteristics	References
Male C57BL/6 mice	End-CCA to side-EJV		1 and 6 weeks	58% at 6 weeks	Recapitulate the features of failing human AVFs	(44)
			0, 1, 7, 14, 21 days	80% at 3 weeks	Not cause significant changes in overall blood pressure	(31)
	End-EJV to end-CCA using the cuff		1, 3, 7 days	100% at 7 days	Recapitulate anatomical and cellular features shown in other species; help to characterize the molecular mechanisms of vascular adaptive changes	(45)
		With left nephrectomy and right upper pole occlusion	7, 14, and 28 days	100% at 28 days	Successful uremic condition; help to silence genes for follow-up studies	(46)
	End-EJV to side-CCA		7, 14, 28 days	88%, 90%, 50% at time points respectively	Similar configuration and features to the most frequently used in human	(28)
			21 days	82% at 21 days	Help to study the role of elastin in AVF remodeling in elastin haplodeficient (eln^+/−^) mice	(47)
			14 days	83% at 14 days	Help to find relaxin receptor deficiency promotes vascular inflammation in RXFP1 knockout (Rxfp1^−/−^) mice model; hard to perform flow measurements and cannulations	(48)
		With 5/6 nephrectomy	2, 4 weeks	62% (CKD), 86% (non-CKD) at 4 weeks	Validation of the role of uremic toxins in AVF stenosis and thrombosis	(49)
	Aortocaval needle puncture		1, 7, 14, 21, 28, 35, 42 days	67% at 42 days	Recapitulate features of AVF maturation and failure in long-term follow up; a simple, safe and powerful tool to study the improvement of AVF outcomes	(50)
Female and male C57BL/6 mice			0, 3, 7, 21, 42 days	25.7% (female), 64.3% (male) at 42 days	Similar dilation and wall thickening during early AVF remodeling and sex difference in AVF patency was shown	(51)
Female wistar rats	End-FA to side-FV		3, 14, 28 days	100% at 28 days	Hemodynamics, maturation and vascular remodeling similar to native fistula	(18)
Sprague–Dawley rats	End-FA to side-FV		between 1 and 4 weeks	100% at all time points	Not lead to lower extremity edema or venous fibrosis; display similar histological features	(52)
Female Sprague–Dawley rats		With adenine diet	0, 7, 14, 21, 42, 63, 84 days	96% at 42 days	Uremic condition; reliable model to test novel therapeutic strategies	(30)
	End-FV to side-FA	With adenine diet	21, 42, 63, 84 days	93.75% at 12 weeks	Recapitulate features of AVF maturation and typical cardiovascular features as in human	(53)
Male Sprague–Dawley rats	End-EJV to end-CCA using the cuff	With 5/6 nephrectomy	7, 28 days	100% at 28 days	An ideal experimental animal model of early and metaphase chronic renal insufficiency for research into intimal hyperplasia	(54)

AVF, arteriovenous fistula; CCA, common carotid artery; CKD, chronic kidney disease; EJV, external jugular vein; FA, femoral artery; FV, femoral vein; RXFP1, RLN/insulin-like peptide family receptor 1.

## 3 The evolutionary history of establishment methods

**Figure 1:**
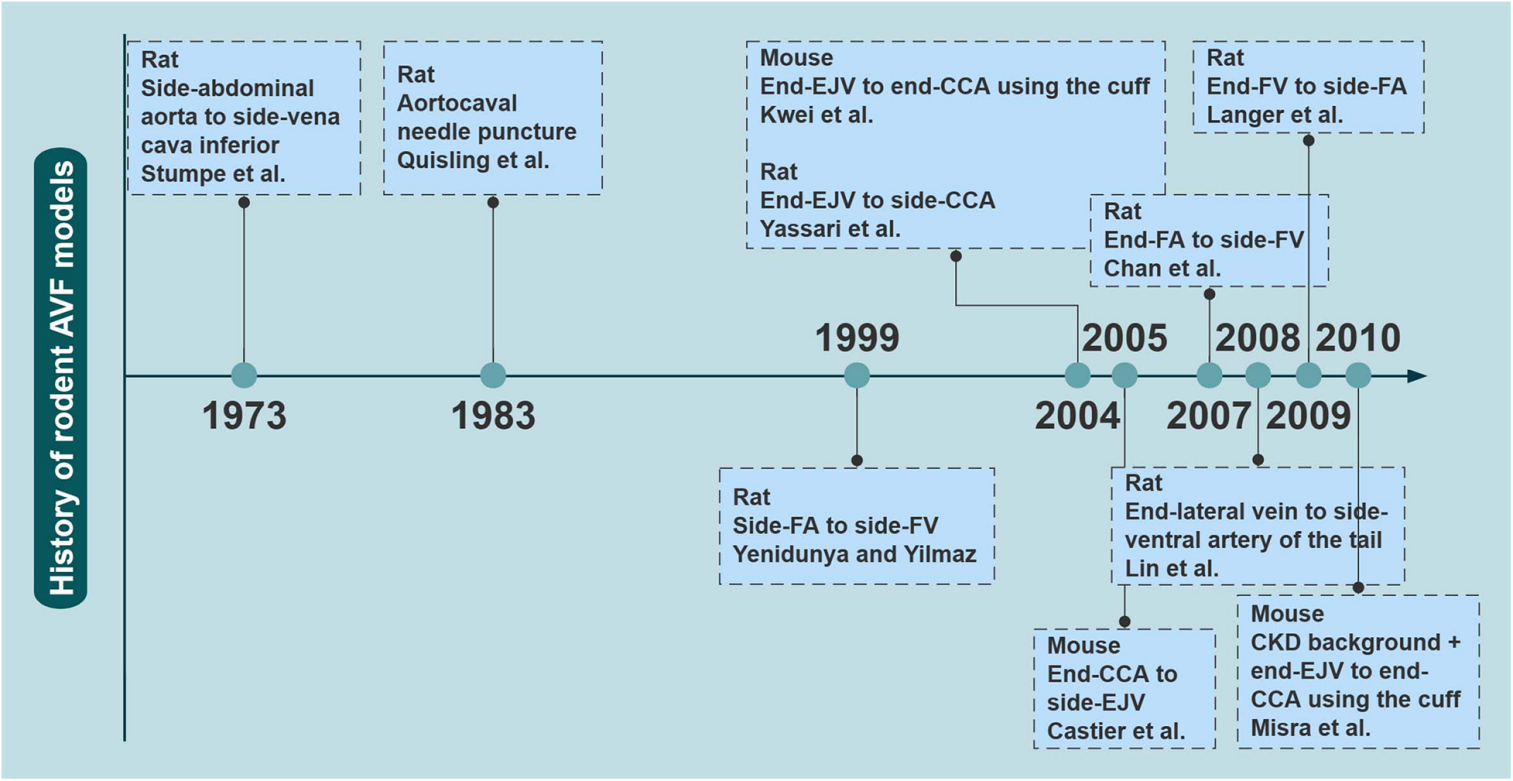
The evolutionary history of rodent AVF models. The earliest reported time points for several major rodent AVF models are shown. AVF, arteriovenous fistulas; CCA, common carotid artery; CKD, chronic kidney disease; EJV, external jugular vein; FA, femoral artery; FV, femoral vein. Created with processon.com.

A variety of rodent models have been explored to study AVF failure owing to the advantages of rodents, such as short reproductive cycle and economy. In particular, as the genome of the mouse has been studied in depth and technological advancements have been made, rodents are a good choice for investigating the molecular mechanisms of disease. There has been a marked increase in the number of related studies over the last two decades in Figure 1.

**Figure 2:**
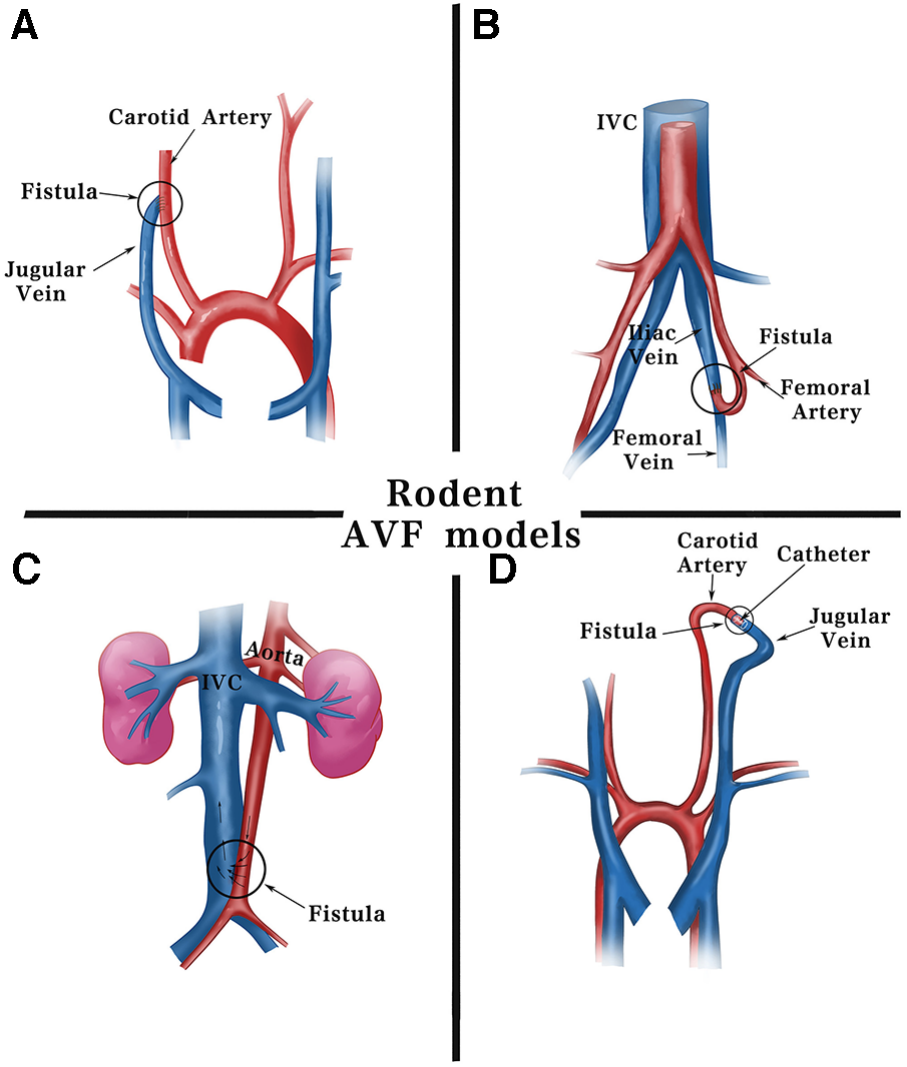
Schematic diagram of surgical methods for fistulas in rodents. Current fistula models in rodents are commonly established using including: (**A**) end-jugular vein to side-carotid artery by surgical suturing. (**B**) End-femoral artery to side-femoral vein. (**C**) Side-aorta to side-inferior vena cava by needle puncturing. (**D**) End-jugular vein to end-carotid artery using cuff technique. IVC, inferior vena cava.

In 1973, Stumpe et al. first created aortic fistulas in Sprague–Dawley rats, but they were mostly used as an experimental model for a long time to study cardiac volume overloading (55). Ten years later, Quisling and Mickle et al. reported the creation of a rat aortocaval fistula by needle puncture to assess histopathological and angiographic changes in the fistula itself, and this classic method is used to date (56). Around 1999, side-to-side anastomoses of femoral vessels for AVF were reported (33). In the following years, it was also reported that fistulas in rodents could be accomplished by anastomosing the femoral vessels in different ways (17, 18, 57). Side-to-end anastomosis of the common carotid artery (CCA) to the external jugular vein (EJV) was developed in rats in 2004, truly characterizing the AVF itself (58). The first mouse AVF model was developed by Kwei et al. in 2004 and was based on end-CCA to end-EJV via the vascular cuff technique (45). Castier et al. created a surgical mouse model to suture the arterial end to the lateral wall of the vein in 2005, which is different from the human AVF (31). In 2008, Lin reported an innovative superficial tail fistula in rats, but it was not successfully ap-plied owing to complications (59). Nowadays, the original methods have been improved, and the combination of CKD pre-induction and AVF structures has been developed in rodents, which can highly simulate disease conditions (46). A comparison of the different methods of creating AVF in rodents is shown in Table 2 for reference. In conclusion, mice and rats are the most widely used rodents to create AVF models; however, consensus on how to induce the model is still lacking. Further improvements are needed in rodent models to stably target the induction of NIH and mimic the failure process (Figure 2).

**Table 2:** Comparison of the advantages and limitations of different rodent AVF models.

Surgical method	Type of AVF		Operative time	Time to neointima formation	Technical success rates	Survival rates	Advantages	Limitations	References
Surgical suturing	Carotid–jugular fistula	End-EJV to side-CCA	45–60 min	2 weeks	67% in the beginning; 97% after training	86% until time to sacrifice	Identical AVF configuration as HD patients; rapid development of NIH; good perfusion and cleanness	Technical challenges; cardiac hypertrophy	(14, 28, 60, 61)
		End-CCA to side-EJV	80 min	1 week; significant at 3 weeks	90%	80%	Significant NIH around the anastomosis; develop NIH in 3 weeks; simulate the hemodynamic to the maximum extent; not cause significant changes in overall blood pressure	Different configuration from HD patients	(13, 62)
		End-CCA to end-IJV		1 month			Pathologic characteristics similar to human	Different configuration from HD patients; not limb vessels; increased cardiac overload	(63)
	Femoral fistula	End-FV to side-FA	47 min (range: 39–55); 27 min (range: 23–56)	≤3weeks	93%	87.5% at 3 weeks; 75% at 6 weeks	Typical features of fistula maturation resemble clinical findings	Massive fibrosis of the media and fusion with the lateral side; subsequent hind limb oedema	(17, 53, 64, 65)
		End-FA to side-FV	57 min (range: 45–73); 32 min (range: 26–41)	≤28 days		83% at 42 days	Native fistula hemodynamics with a high flow and low pressure	Not superficial	(18, 30)
	Aortic fistula	Side-abdominal aorta to side-vena cava inferior		≤4 weeks		90% at 2 weeks	Typical NIH similar to patients	Severe ventricular hypertrophy and heart failure	(66)
		End-renal vein to end-aorta	45 min	1 week		93.3% at 4 weeks	Rapid and significant development of NIH	Location in the abdominal cavity; lack of CKD; ventricular hypertrophy and heart failure	(67)
	Tail fistula	End-lateral tail vein to side-ventral tail artery		≤28 days	80%	100% at 28 days	Superficial visibility; allowing for subcutaneous treatment and monitoring studies	Histological and structural differences; multiple complications	(59)
Needle puncture	Aortocaval fistula	Side-abdominal aorta to side-vena cava inferior	<10 min	21 days	83% after training	90% at 4 weeks; 50% at 8 weeks	Simple and clean; rapid; relatively sterile; high modelling stability and reproducibility	Local haemodynamics vary considerably; lead to cardiac failure	(50, 68)
Cuff technique	Carotid–jugular fistula	End-CCA to end-EJV	40–60 min	1 week; significant at 4 weeks	65%; 100%	100% at 7 days	Minimal invasive; higher success rate without microsurgical suture; low incidence of blood leakage; less likely to cause thrombosis	Different configuration and presence of an intravascular catheter	(45, 54, 63)

AVF, arteriovenous fistula; CCA, common carotid artery; CKD, chronic kidney disease; EJV, external jugular vein; FA, femoral artery; FV, femoral vein; HD, hemodialysis; IJV, internal jugular vein; NIH, neointimal hyperplasia.

## 4 Rodent AVF models

### 4.1 Pre-induction of stable CKD in rodent AVF model

Chronic renal dysfunction has been suggested to have an impact on the systemic environment and pathogenesis of AVF failure (69, 70). In patients with CKD, the number of circulating endothelial progenitor cells is reduced, and their adherence, outgrowth potential, and anti-thrombogenicity are decreased (71). A clinical study confirmed pre-existing inflammatory and oxidative markers in the veins of patients with CKD (72). The accumulation of uremic toxins impairs mitochondrial function and elevates oxidative stress levels in the body (73). The systemic effects of CKD promote cytokine pro-duction and proliferation and migration of vascular SMCs, which are involved in intimal hyperplasia formation (74). Undoubtedly, this complex disease context is important for finding appropriate animal models to simulate a hypothesis and study a scientific problem.

Existing experimental rodent models of CKD have been constructed by inducing an appropriate pathology based on its complex and diverse etiology (Table 3).

**Table 3:** Major CKD models in rodents.

CKD Models	Methods
Remnant kidney model	Unilateral nephrectomy and partial infarction of the remaining kidney (46)
	5/6 subtotal nephrectomy (52, 63)
Adenine-induced	0.75% adenine-containing diet for 4 weeks (66)
Diabetic nephropathy	Nicotinamide, alloxan and (or) streptozotocin infusion; high-fat diet; gene modified (75)
Hypertension-induced	Angiotensin II infusion; spontaneously hypertensive rats with unilateral nephrectomy (76)
Primary glomerular nephropathy	Adriamycin or uromycin injection; transgenic mice (77, 78)
Secondary nephrotic syndrome	Lupus-prone mouse strains; transgenic mice (79)
Hereditary nephritis	Gene engineered mouse (80)

The successful induction of CKD in rodent model is mostly verified by significant changes in biochemical index levels compared to the normal group, such as an approximately two-fold increase in serum urea nitrogen and creatinine, in addition to the detection of proteinuria or renal tissue sections with significant collagen deposition and other characteristics of kidney injury, for which there are no specific levels to judge. Subtotal nephrectomy is a more frequently used induction approach of CKD complementary to the AVF model, although this method has a high mortality rate of approximately 40% (81–49). Two-step five-sixth partial nephrectomy was used in a study on CKD-promoted AVF failure by Ding et al., in which approximately two-thirds of the left kidney was first removed 4 weeks before establishing AVF, and the right kidney was removed after feeding the mice a 6% protein chow for 1 week, followed by a 40% protein chow to lower mortality rate (83). In another study using 5/6 nephrectomy combined with a jugular AVF rat model, the animals were healthy except for one rat that died during anesthesia, and 3 of 17 developed AVF stenosis after 1 week, with significantly higher urea and creatinine levels in the model group at 3 weeks (39). Misra et al. successfully induced venous stenosis using left nephrectomy and right upper pole occlusion with AVF placement after 4 weeks (46, 84). However, an infarct-induced remnant kidney model may be accompanied by hypertension, proteinuria, and glomerulosclerosis. Notably, the current use of chronic adenine-containing diets to induce uremic models can produce both stable renal damage and the induction of cardiovascular diseases, such as CKD in humans (85). Blood urea nitrogen and serum creatinine were reported to be elevated in this model after 4 weeks of adenine feeding, closely resembling the clinical scenario (60, 66). This method is only available for rats or mice, which is an advantage of rodent animal models (30, 86). Due to the high individual variability in uremia, the lack of consistency in renal unit mass reduction makes it difficult to standardize, which is a direction worth exploring. It is certain that a high degree of consistency in the baseline characteristics of the rodents, modeling methods and timing to harvest can reduce the error. The mainstream of renal failure models that have been validated to cause renal injury are the remnant kidney model and adenine feeding, which is exactly what we recommend.

In addition, both diabetic nephropathy and hypertension-induced renal damage are important causes of ESRD. There is no ideal rodent model for diabetic nephropathy; however, administering alloxan and streptozotocin (glucose analogs) or high-fat diet, spontaneous development based on genetic background, and genetic engineering modifications are some reported modalities which can be used for establishing such models (41, 75). Models of hypertension and renal damage can be induced by angiotensin II injections for several weeks, and by unilateral nephrectomy in spontaneously hypertensive rats (76). Other CKDs such as lupus nephritis, polycystic kidney disease, and IgA nephropathy can be mimicked in genetically modified mice, but these models are costly, difficult to construct, and progress slowly, and even develop ESRD less frequently (79–77). Injection of adriamycin and puromycin can successfully induce glomerulosclerosis in rodent models (78). All these models can provide some ideas for establishing a stable CKD milieu in rodent AVF models.

### 4.2 Surgical suturing methods in rodent AVF models

#### 4.2.1 Carotid–jugular fistula

Carotid-jugular fistula models mimic human peripheral fistulas and are extensively used in studies on AVF failure mechanisms. To date, the most widely accepted method in rodents is anastomosis from the end of the EJV to the ipsilateral side of the CCA, with the vein acting as an outflow tract, which has an identical AVF configuration as the clinical application (44). The incision in the arterial wall of mice is only 1 mm; therefore, technically it is highly demanding (47). Yassari et al. first observed this type of fistula in rats at five time points over 90 days and found that the hemodynamics showed a stable high flow and low resistance state immediately after fistula formation, whereas after 21 days, the angiographic and histological presentation of the fistula stabilized (58). Analysis of fistula sections from this model demonstrated that endothelial molecular changes resemble those observed in humans (87). The same method was applied to mice, and the patency rates on days 7, 14, and 28 were 88%, 90%, and 50%, respectively. The success rate of the procedure was up to 97% after adequate practice (28, 61). In contrast, Castier et al. created an end-CCA to side-EJV anastomosis in mice, with a 3-week patency rate of 80% for the fistula, and significant NIH induction at the anastomosis (31). Another study reported an overall perioperative mortality rate of 20%, mainly related to anesthesia or postoperative bleeding (13). Significant thickening of the arterial wall at the anastomosis site was observed at 1 week postoperatively, with a 100% and 33% patency rate of the fistula at 3 and 4 weeks, respectively. In addition, it did not cause significant changes in overall blood pressure (13, 31). Liang et al. performed direct end-to-end anastomosis of the CCA and internal jugular vein with interrupted sutures in mice using 11-0 nylon sutures and observed more severe NIH in the venous portion near the anastomosis (63, 88). It is considered that end-EJV to side-CCA able to make the vein arterialized would be more suitable for investigational study and drug compound test, due to maximizing similarity to clinical. It has not been reported which is better, the end-to-end cuff model or end-to-side anastomosis, but the former can reduce the trauma produced by suturing. CCA to EJV is more widely used than CCA to IJV, because CCA to IJV requires connection to the thinner vein (IJV), which is located anatomically closer to the CCA and is not conducive to modeling the angle formed during AVF.

#### 4.2.2 Femoral fistula

Initially, a side-to-side anastomosis was made between the common femoral artery (FA) and femoral vein (FV) under microsurgical magnification to create a femoral AVF (33, 57). Subsequently, another surgical procedure of end-to-side fistula in rat was developed, in which an inguinal incision was made, followed by dissection of the distal FA and anastomoses of the end to the lateral wall incision of the FV. Increased MMP expression during AVF maturation was demonstrated in this model (18). The end of the FV was sutured to the side of the FA to form a femoral fistula, with an anastomotic length of approximately 2.5 mm. The suture was more consistent in structure than that used in human AVF (64, 89). In a study by Langer, the average operation time was reduced to 27 min, all 15 rats survived, and the fistula patency rate was 93% at 12 weeks (17, 53). However, histological findings showing massive fibrosis of the media and fusion with the lateral side were once reported, and the subsequent hind limb edema in the animal suggested its limitations (90). Croatt suggested that such complications could be avoided by not ligating both the femoral vein above the AVF and the superficial epigastric vein after the creation of the femoral AVF (52). Moreover, a mouse model for studying HD-related limb dysfunction through direct anastomosis of the iliac artery and vein has recently been reported, with an approximately 80% patency rate in surviving mice (34). However, the iliac veins in this model are part of the deep venous system and differ from superficial AVF in clinical patients (18).

#### 4.2.3 Aortic fistula

The fistula can also be constructed by surgical anastomosis of adjacent arteries and veins in a side-to-side manner after vessel incision, such as the descending aorta and inferior vena cava (66). To rapidly and significantly induce NIH, the distal renal vein can be anastomosed to the abdominal aorta, and intraoperative ligation of the renal vein for several minutes was optionally attempted to create an aortic fistula in rats, but it later induced severe ventricular hypertrophy and heart failure (67, 91).

#### 4.2.4 Tail fistula

In 2008, Lin et al. first reported tail fistula in a rat model (59). It is characterized by superficial visibility and can therefore be used for subcutaneous treatment and monitoring studies. For this purpose, a more superficial lateral vein was selected and its end was anastomosed to the side of the ventral artery of the rat's tail. Five fistulae were successfully operated on, and dilated veins were visible through the skin after 28 days, four of which showed NIH on histological analysis. However, this model is very different in structure from that of humans, and the utility and validity of the model are not guaranteed owing to multiple complications, particularly adhesions of scar tissue followed by local compression (92).

### 4.3 Rodent AVF models created using aortocaval needle puncture

A simple and clean method to create a fistula is by puncturing the aorta into the vena cava using anatomical features. The general procedure involves exposing the vena cava and abdominal aorta retroperitoneally, cross-clamping them, and creating a venous incision through which the needle penetrates the opposite vessel wall. Finally, the venous incision is closed, the clamps are removed, and an aortocaval fistula is formed without additional suturing when arterial pulsatile flow is evident in the inferior vena cava at the puncture site (50, 93). Mickle et al. reported histopathologic and angiographic assessments of aortocaval fistulas at various stages, from 1 day to 6 months (56). According to a study by Yamamoto, the model showed maturation characteristics of a dilated and thickened AVF with increased blood flow in 75% of mice at day 21, whereas 33% of fistula stenoses failed at day 42, which is very similar to the human AVF maturation process (50). This model is significant for the study of the biological process of vascular remodeling and the mechanism of AVF dysfunction after venous injury. However, central vessels are characterized by high-flow, which may differ from more peripheral processes, and are located deeper, making them less amenable to superficial observation.

### 4.4 Rodent AVF models created using the cuff technique

As the outer diameter of the carotid artery is approximately 2–3 mm in rats and 0.3–0.5 mm in mice, end-to-end suturing of the proximal CCA to the distal EJV can be performed; however, this procedure is challenging. The cuff technique can be used to simplify small vessel anastomosis (94). In the study by Misra et al., the mortality rate on postoperative day 1 in mice with end-to-end carotid-jugular fistulas was 20% (95). The technique requires the preparation of a small cannula with the appropriate diameter and length, which has been reported in mice with an internal and outer diameter of 0.2–0.28 mm and 0.4–0.61 mm, respectively, while in rats a disposable venous indwelling needle catheter is used (45, 46, 54). The CCA is detached and cut, the severed end of the artery is led through the catheter, and the arterial blood vessel wall is turned out and wrapped around the catheter, followed by the insertion of the cannula containing the arterial end into the jugular vein, secured with a suture around the ligature. This type of vascular anastomosis is less invasive and has a low incidence of blood leakage with a lower likelihood of causing thrombosis, as it only approaches the artery adventitia (45, 54). However, a catheter is placed inside the vessel, and the extent to which it mimics a human AVF is limited.

## 5 Evaluation of rodent models after AVF placement

**Figure 3:**
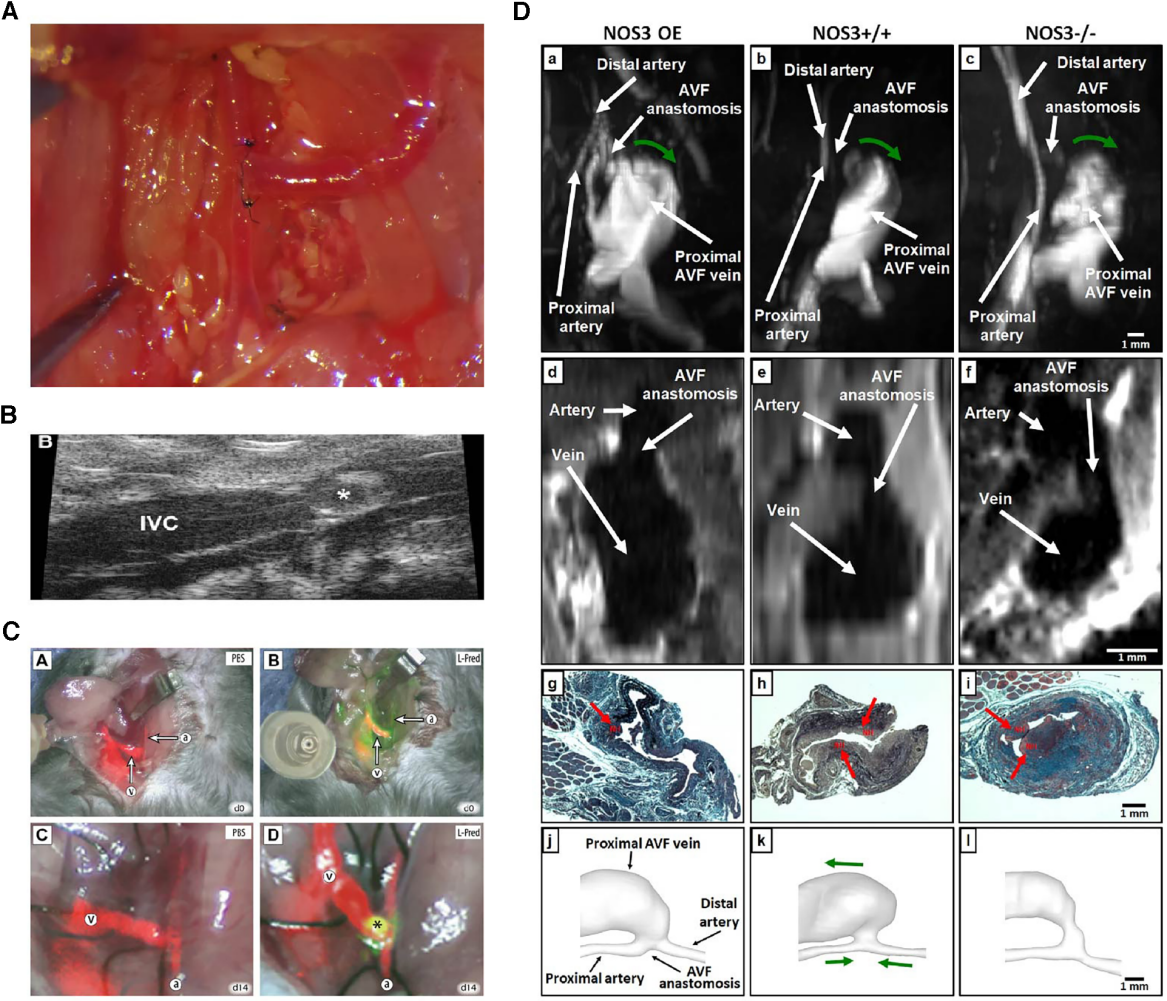
Various evaluation methods on fistulas. (**A**) Macroscopic view of side-to-end carotid-jugular fistula post-operation. (**B**) Assessment of the aortocaval fistula by ultrasound by Dardik et al. (97). (**C**) Near-infrared fluoroscopy images of patency of fistula by Wong et al. (98). (**D**) Representative images of magnetic resonance imaging, histology and lumen geometrical models showing fistula characteristics by Daniel et al. (99).

Apart from using appropriate approaches to confirm the success of the models, assessing the pathophysiological development of the fistula after placement is another crucial aspect. Important evaluations usually require the determination of patency, changes in hemodynamic features, and histopathological examination of the fistula (96) (Figure 3).

Hemodynamic alterations are strongly associated with the development of adaptive vascular remodeling and dialysis access dysfunction, the observations of which include blood flow patterns, pressure and velocity analysis, and WSS (100). Due to the small diameter of the blood vessels in rodent models, there may be some technical difficulties in studying intravascular blood flow, which often involves the use of a perivascular flow probe or various imaging techniques (62). Doppler ultrasound has the advantage of easy handling and real-time non-invasive imaging, which can be used to confirm vessel patency, accurately measure changes in transit-time flow, velocity tracings, inner diameter of vessels, and provide values to calculate WSS (17, 101). Magnetic resonance imaging (MRI) combined with angiography allows an accurate assessment of the geometry and flow changes at multiple locations and time points in the blood vessels and has been shown to be a good predictor of AVF maturation (45, 58, 64, 65). Recently, non-contrast MRI imaging has been used to reconstruct the three-dimensional lumen structure at a higher resolution (orders of 0.5 µm and 0.1 ms) in a mouse model to reveal the real-time spatiotemporal characteristics of local wall hemodynamics in AVF by computational fluid dynamic (CFD) simulations. However, the correlation between early *in vivo* hemodynamic parameter changes, late lumen vascular remodelling, and NIH development has not been finely analyzed (100). Currently, an MRI-based fluid-structure interaction (FSI) study in a mouse AVF model was introduced for additional comprehensive assess of hemodynamic and wall mechanics parameters (99). Additionally, dextranated magnetofluorescent nanoparticles [CLIO-VT680 (cross-linked iron oxide-VivoTag680)] can be deposited on pathological endothelial cells near the AVF anastomosis, enabling *in vivo* observation and prediction of subsequent inflow tract NIH using intravital microscopy or MRI imaging (102). With infrared fluorescence imaging, direct AVF patency can be assessed using video imaging with the aid of a fluorescence-assisted resection and exploration imaging system (28, 98). Live animal *in vivo* evaluations are a great choice that enable longitudinal follow-up imaging and can minimize animal sacrifice.

Histological staining of vessel sections after perfusion-fixed outflow and control veins allows for the assessment of morphological changes. Common staining includes hematoxylin and eosin, which allows the measurement of fistula neointima and media thickness, statistical patency rates, and thrombosis. Masson's trichrome and Verhoeff–Van Gieson staining are useful for evaluating the continuity and integrity of smooth muscle fibers, intercellular fibers, and collagen (14, 103). With a deeper understanding of the mechanisms of AVF failure, the assessment of oxidative stress markers and indicators of inflammation is also a good complement (104). Immunohistochemical assessment helps in target protein localization; western blotting and reverse transcriptase polymerase chain reaction are also essential tools for determining the expression of molecules in specific signalling pathway mechanisms (44). Except traditional methods, some advancement in molecular assays (for instance, spatial multiomics: at transcriptional, translational, metabolic, and epigenetic levels and multiplex fluorescent protein assay) can be considered for future investigation of the microenvironment of AVF failure and how AVF interacts with it surrounding tissue/cells. However, due to the limited amount of available venous tissue in rodent models, an adequate sample size is needed for statistical analysis of such a protein (44).

## 6 Discussion

Currently, AVF is the gold standard for dialysis access, maintaining prescribed dialysis treatment for more than 3 million patients with CKD progressing to ESRD worldwide (1, 2). Unfortunately, fistula maturation failure and poor patency remains problematic for the administration of HD; thus, suitable animal models are needed to simulate and study human diseases (105, 106).

Despite some disease research limitations and limited translational value, rodent models have helped to a great extent in many aspects. On the one hand, owing to advances in transgenic and sequencing technologies, specific biomarkers and potential therapeutic targets can be identified using current rodent models. A few specifically modified mice were used to study the important role of changes in gene expression in AVF failure (47, 107, 108). For example, researchers constructed heme oxygenase-1 (HO-1) gene-deficient mice and demonstrated that their vascular access is more likely to be dysfunctional (109). Knockout of monocyte chemoattractant protein-1 (MCP-1) in a mouse was found to be beneficial in increasing the patency of fistula (110). Adenoviral vector-mediated gene delivery upregulated HO-1 in a mice AVF model of CKD, showing beneficial effects (111). On the other hand, rodent AVF models are used to elucidate the pathogenesis and underlying mechanisms including cellular events, morphology, and pathologic changes behind the loss of AVF function. Liang et al. demonstrated that 50% of SMCs in AVF neointima formation originated from arterial anastomoses using techniques such as fluorescent protein gene labeling in a mouse AVF model (112). Evaluating hemodynamic changes and NIH in rodents by using various approaches may provide important information about the physiology and pharmacology of AVF (62, 103). Furthermore, the role and effects of drugs such as sulodexide (113), simvastatin (84), rosuvastatin (42) have been studied in this model. Use of these drugs along with the local administration of endovascular devices or antigen-coated nanoparticles can be attempted, offering the prospect of treatment of AVF failure. Finally, the establishment of a mixed model of disease in rodents, such as those with CKD or diabetes, is more helpful in exploring the impact and mechanisms of clinical comorbidities.

As mentioned previously, several recently established representative methods of rodent AVF models are reliable and can provide good references to contribute to research. The modified end-EJV to side-CCA rodent AVF model by Misra et al. had the highest degree of clinical peripheral mimicry, showing the complete pathophysiological process of AVF maturation to NIH, and the fistula site is easily accessible for local administration and follow-up with ultrasound (60, 114). Interestingly, an attempt to anastomose the right end-EJV to the left side-CCA could provide an outflow vein length of 10 mm; therefore, this model could be applied for studying restenosis after angioplasty (14). However, these models are technically challenging and can be influenced by the experience and microsurgical skills of the researchers in different laboratories. The needling method commonly used by Dardik et al. greatly reduces variations due to operator factors and has the advantages of being simple, rapid, and relatively sterile (25, 50, 51). It has also been reported that the diameter of the fistula can be controlled by selecting different needle sizes, thereby achieving high modelling stability and reproducibility (97). The limitation is that the local hemodynamics of aortocaval fistulas vary considerably and may easily lead to ventricular hypertrophy and cardiac failure (68). Narrower vessel diameters, lack of valves in veins, and different evolutionary processes of blood coagulation can lead to faster thrombosis in rodent models (17). Thrombotic lesions always occurs secondary to AVF restenosis in humans, and we're supposed to exclude mice with thrombus-obstructed vessels from AVF models, focusing on the pathology of intimal hyperplasia. Another limitation is that young male animals are more often used due to their high tolerance and long probability and duration of survival after operation, however, older AVF rodent models may have different study results since most of CKD patients in late middle to elder age rather than in young population.

With the maturity and improvement of AVF rodent model, the future development of its related research is worth to be expected. Firstly, female has been reported to have lower AVF patency and durability compared with men. This difference may be caused by lower velocity, small vessel size, lower shear stress, sex hormone difference (115). It may also be due to sex-specific genes/proteins involved in thrombosis/inflammatory/proliferative pathways during AVF remodeling (116–118), and this difference deserves the continued attention of researchers. Second, this disease is often an altered systemic environmental state accompanied by changes in distant vascular bed and other organ's function (e.g., heart, kidneys, and brain), especially as cardiovascular events account for more than half of the mortality in patients with ESRD (119). Our studies tend to focus only on the local area, and it is also important to understand the regional and systemic hemodynamic changes in each murine AVF configurations. Moreover, the current AVF model is highly influenced by the surgical operator, and the stability and homogeneity of the model should be improved in the future. Various innovative vascular anastomosis devices (which has already been applied in the clinic) can be considered, besides, the stable establishment and standards regarding the CKD model are yet to be determined.

Overall, rodents have played an important role in advancing AVF-related research in recent years and will continue to serve as a transitional tool for preclinical model translation to simulate diseases and help reveal disease features.
